# Cigarette smoke induces mitochondrial DNA damage and activates cGAS-STING pathway: application to a biomarker for atherosclerosis

**DOI:** 10.1042/CS20220525

**Published:** 2023-01-24

**Authors:** Keitaro Ueda, Chiemi Sakai, Takafumi Ishida, Kosuke Morita, Yusuke Kobayashi, Yasunori Horikoshi, Akiko Baba, Yuma Okazaki, Masao Yoshizumi, Satoshi Tashiro, Mari Ishida

**Affiliations:** 1 Department of Cardiovascular Physiology and Medicine, Graduate School of Biomedical and Health Sciences, Hiroshima University, Hiroshima 734-8551, Japan; 2 Department of Cardiovascular Medicine, Fukushima Medical University, Fukushima 960-1295, Japan; 3 Department of Cardiovascular Medicine, Graduate School of Biomedical and Health Sciences, Hiroshima University, Hiroshima 734-8551, Japan; 4 Department of Cellular Biology, Research Institute for Radiation Biology and Medicine, Hiroshima University, Hiroshima 734-8551, Japan

**Keywords:** biomarker, cell-free DNA, cGAS-STING, DNA damage, mitochondria

## Abstract

Cigarette smoking is a major risk factor for atherosclerosis. We previously reported that DNA damage was accumulated in atherosclerotic plaque, and was increased in human mononuclear cells by smoking. As vascular endothelial cells are known to modulate inflammation, we investigated the mechanism by which smoking activates innate immunity in endothelial cells focusing on DNA damage. Furthermore, we sought to characterize the plasma level of cell-free DNA (cfDNA), a result of mitochondrial and/or genomic DNA damage, as a biomarker for atherosclerosis. Cigarette smoke extract (CSE) increased DNA damage in the nucleus and mitochondria in human endothelial cells. Mitochondrial damage induced minority mitochondrial outer membrane permeabilization, which was insufficient for cell death but instead led to nuclear DNA damage. DNA fragments, derived from the nucleus and mitochondria, were accumulated in the cytosol, and caused a persistent increase in IL-6 mRNA expression via the cyclic GMP-AMP synthase (cGAS)-stimulator of interferon genes (STING) pathway. cfDNA, quantified with quantitative PCR in culture medium was increased by CSE. Consistent with *in vitro* results, plasma mitochondrial cfDNA (mt-cfDNA) and nuclear cfDNA (n-cfDNA) were increased in young healthy smokers compared with age-matched nonsmokers. Additionally, both mt-cfDNA and n-cfDNA were significantly increased in patients with atherosclerosis compared with the normal controls. Our multivariate analysis revealed that only mt-cfDNA predicted the risk of atherosclerosis. In conclusion, accumulated cytosolic DNA caused by cigarette smoke and the resultant activation of the cGAS-STING pathway may be a mechanism of atherosclerosis development. The plasma level of mt-cfDNA, possibly as a result of DNA damage, may be a useful biomarker for atherosclerosis.

## Introduction

Cigarette smoking is a known risk factor for atherosclerosis. Thousands of chemicals in cigarette smoke have been reported to contribute to the development of cardiovascular diseases through various mechanisms, such as inflammation, enhanced hemostasis, endothelial dysfunction, and increased heart rate [1].

There are several reports suggesting that DNA damage is involved in the development of atherosclerosis. Patients with progeroid syndromes, e.g., Werner’s syndrome and Hutchinson–Gilford progeria syndrome, which result from abnormal DNA repair and subsequent DNA damage, develop atherosclerotic disease at a young age [2]. In addition, the relative risk of ischemic heart disease is increased after radiation therapy for breast cancer [3] and we have previously reported that DNA damage was accumulated in atherosclerotic lesions [4]. In terms of smoking, nuclear DNA damage to peripheral mononuclear cells was higher in smokers compared with nonsmokers [4]. However, the molecular mechanisms whereby smoking-related DNA damage causes atherosclerosis remain unclear.

The accumulation of DNA damage is considered to be one of the hallmarks of cellular senescence. Senescent cells express a variety of secretory factors called senescence-associated secretory phenotype (SASP), including inflammatory cytokines, growth factors, and chemokines. In addition, recent studies have reported the cytosolic DNA-sensing pathway as a mechanism by which DNA damage induces inflammation [5]. Innate immunity utilizes nucleic acid sensors to detect RNA and DNA viruses, and triggers inflammation; these sensors have been reported to also detect self-DNA [5]. As inflammation plays a central role in all phases of the atherosclerotic process [6], cytosolic DNA and DNA sensors may be involved in the development of atherosclerosis.

Cytosolic DNA is incorporated into extracellular vesicles [7,8]; thus, cell-free DNA (cfDNA), which is present in human peripheral blood, has already had a significant impact on prenatal medicine and is also attracting attention in cancer, transplant medicine, and other areas [9,10]. Endothelial dysfunction is observed from the early stages of atherosclerosis. As blood vessels are the largest organ in the human body and the vascular endothelium is the organ closest to the blood, it has been reported that cfDNA in healthy individuals is released mostly from vascular endothelial cells, except for blood cells such as white blood cells [11], as a result of their damage or cell death. Thus, cfDNA could reflect the pathological condition of the vascular endothelium. As endothelial dysfunction is involved in the pathogenesis of atherosclerosis, it is possible that cfDNA is increased at an early stage of this disease. Currently, flow-mediated dilation is used to evaluate endothelial function, and ultrasonography is used to diagnose atherosclerosis of the carotid artery. It would be very useful to have a biomarker that can detect the genomic and biological damage of endothelial cells in atherosclerosis at an earlier stage without special techniques.

In the present study, to elucidate the mechanisms of cigarette smoke-induced inflammation that are central to the pathogenesis of atherosclerosis, we investigated the effect of cigarette smoke extract (CSE) on both nuclear and mitochondrial DNA damage, and the subsequent cellular response in endothelial cells. To further investigate whether cfDNA in blood could reflect the smoking status and be a new biomarker reflecting the presence and degree of atherosclerosis, we measured cfDNA in the blood of patients with atherosclerosis.

## Methods

### Cell culture

Human umbilical vein endothelial cells (HUVECs) and human aortic smooth muscle cells (HASMs) were purchased from CAMBREX Corporation. The cells were cultured in a basal medium containing the specific growth supplements recommended by the manufacturer. Cells at passages 5–9 were used.

### Preparation of CSE

CSE was prepared in similar method as in the previous report [12]. Briefly, CSE was prepared by dissolving the smoke of eight cigarettes (hi-lite) in 15 ml of PBS and stored at −80°C before use. CSE was sterilized by a 0.22-μm filter (Merck, Germany). A small difference in activity between CSE samples prepared on different days was adjusted after determining cytotoxic activity on HASMs with Cell Counting Kit-8 (Dojindo, Japan). Final concentration of CSE was 0.5% of the medium.

### Immunofluorescent analysis

Immunofluorescent staining was performed using phosphorylated histone H2AX (γH2AX) antibody (Millipore, MA, U.S.A.), 8-hydroxy-2′-deoxyguanosine (8-OHdG) antibody (Bioss, MA, U.S.A.), BAX antibody (Santa Cruz, CA, U.S.A.), caspase-activated DNase (CAD) antibody (Santa Cruz, CA, U.S.A.), double-strand (ds)DNA antibody (Santa Cruz, CA, U.S.A.), phospho-TANK binding kinase 1 (p-TBK1) antibody (Cell Signaling, MA, U.S.A.), and phospho-NF-κB p65 (p-p65) antibody (Cell Signaling, MA, U.S.A.) as previously described [4]. Briefly, cells were fixed with 4% paraformaldehyde and permeabilized with Triton X-100. The cells were incubated with γH2AX antibody, 8-OHdG antibody, BAX antibody, CAD antibody, dsDNA antibody, P-TBK1 antibody, or p-p65 antibody for 30 min at 37°C, and then incubated with Cy3-conjugated secondary antibody or FITC-conjugated secondary antibody for 30 min at 37°C. The nuclei were stained with 4’,6-diamidino-2-phenylindole (DAPI). Mitochondria were stained with MitoTracker™ Red CMXRos (Life Technologies, Varlsbad, CA, U.S.A.) for 45 min at 37°C, in 5% CO_2_. Control experiments were performed using normal IgG for each antibody to confirm that the staining was specific. (Supplementary Figure 1A–D).

The samples were assessed with an Axio Imager Z2 microscope (Carl Zeiss, Germany), equipped with the Metafer4 software (MetaSystems, Germany), a fluorescence microscope BZ-X700 (KEYENCE Co., Osaka, Japan), or an LSM780 confocal laser-scanning microscope (Carl Zeiss, Germany). γH2AX foci were automatically counted in at least 500 cells. The number of γH2AX foci was divided by the number of total cells and expressed as γH2AX foci/cell. For BAX staining, cells with more than ten foci in five different areas of the slide were counted [13]. For 8-OHdG staining, the average intensity per cell in the nucleus and cytosol was calculated by NIH image in five different regions of the slide [14,15]. In the same way, for CAD staining, the average intensity per cell in the nucleus was calculated in five different regions of the slide. For p-TBK1, the average intensity per cell in the cytosol was calculated in three different regions of the slide. For p-p65, the average intensity per cell in the nucleus was calculated in four different regions of the slide. Also, the intensity in cytosolic region was measured for dsDNA by NIH image, largely as described previously [16]. Briefly, the outlines of the cells were traced and their dsDNA intensity were measured (mean intensity multiplied by the area, defined as X), and the outline of the nucleus and their dsDNA intensity were measured (mean intensity multiplied by the area, defined as Y) by FIJI software. The cytosolic dsDNA intensity was calculated as X–Y.

### Evaluation of mitochondrial membrane potential

CSE-stimulated changes in the mitochondrial membrane potential were assessed by JC-1 MitoMP Detection Kit (Dojindo, Japan) following the manufacturer’s protocol. The red (polarized) fluorescence in excitation/emission (535/595 nm) and green (depolarized) fluorescence in excitation /emission (485/535 nm) was measured by Varioskan Flash (Thermo Scientific, U.S.A.) and the ratio of red fluorescence divided by that of green fluorescence was obtained. JC-1 exhibits red fluorescence in normal conditions but green fluorescence when the mitochondrial membrane potential is down-regulated.

### Western blot analysis

Western blot was performed as described previously [17].

### RNA preparation and real-time RT-PCR analysis

RNA preparation and real-time RT-PCR analysis were performed as previously reported [18]. Briefly, total RNA was isolated from cells using TRIzol (Invitrogen, Carlsbad, CA, U.S.A.) according to the manufacturer’s instruction and Ethachinmate (NIPPON GENE, Toyama, Japan) to improve RNA precipitation. Two microgram of RNA was reverse-transcribed into cDNA with random primers using ReverTra Ace (TOYOBO, Japan) as described in the manufacturer’s protocol. Real-time RT-PCR analysis was performed using StepOnePlus Real-Time PCR System (Applied Biosystems, U.S.A.) and THUNDERBIRD SYBR qPCR Mix (TOYOBO, Japan) to detect levels of the mRNAs for interleukin 6 (IL-6), interleukin 1α (IL-1α), monocyte chemoattractant molecule 1 (MCP-1), Interferon-β (IFN-β), and 18s ribosomal RNA. The sequences are shown in Supplementary Table S1.

### Measurement of cGAMP

cGAMP concentration in cell lysates was measured using a 2’3’-cGAMP ELISA Kit (Cayman Chemical, U.S.A.) according to the manufacturer’s protocol. The result was normalized by total protein concentration as described previously [16].

### Gene silencing by small-interfering RNA

HUVECs were transfected with small-interfering RNA (siRNA) against human cyclic GMP-AMP synthase (cGAS) (s41746 or s41748, ambion, U.S.A.), BAX (s531483, ambion, U.S.A.), or AllStars Negative Control siRNA (QIAGEN, U.S.A.) using Lipofectamine-RNAiMax (Invitrogen, U.S.A.) according to the manufacturer’s protocol. The sequences are shown in Supplementary Table S2.

### Cytosolic DNA isolation and quantification

Cytosolic DNA isolation and quantification were performed largely as described previously [19]. Briefly, HUVECs were harvested using 0.05 w/v% Trypsin-0.53 mmol/l DTA·4Na solution, divided into two equal aliquots and centrifuged at 1000 rpm for 3 min to precipitate cells. One aliquot was resuspended in 200 μl of 50 μmol/l NaOH and boiled for 30 min to solubilize DNA. The supernatants contained whole-cell DNA. Twenty microliter of 1 mol/l Tris-HCl pH 8 was added to neutralize the pH, and these extracts served as normalization controls for cytosolic DNA. The other aliquot was resuspended in 200 μl buffer containing 150 mmol/l NaCl, 50 mmol/l HEPES pH 7.4, and 25 μg/ml digitonin (TCI, JAPAN). The homogenates were incubated end over end for 10 min, then centrifuged at 17000 ***g*** for 10 min and the supernatants were transferred to fresh tubes. The supernatants were cytosolic preparations free of nuclear, mitochondrial, and endoplasmic reticulum contamination. Then, DNA was isolated from these pure cytosolic preparations using QIAQuick Nucleotide Removal Columns (QIAGEN). Quantitative PCR was performed on both whole-cell extracts and cytosolic fractions using nuclear DNA (nDNA) primers (β-globin) or mitochondrial DNA (mtDNA) primers (NADH dehydrogenase 1; NADH1), and the Ct values obtained for whole-cell extracts served as normalization controls for the Ct values obtained from the cytosolic fractions. The sequences are shown in Supplementary Table S1.

### Measurement of cfDNA in the culture medium

After HUVECs were incubated with CSE for 2 days, the culture medium was transferred to microtubes and centrifuged (1000× ***g***, 10 min) to remove the cell debris. For DNA isolation from culture medium, the NucleoSpin cfDNA XS (TAKARA, JAPAN) was used with a starting sample volume of 720 µl and an elution volume of 20 µl according to the manufacturer’s protocol. We also extracted whole-cell DNA using the method described above. Quantitative PCR was performed on both cfDNA in cultured medium and whole-cell DNA using nDNA primers (β-globin) and mtDNA primers (NADH1), and the Ct values obtained for whole-cell DNA served as normalization controls for the Ct values obtained from cfDNA in cultured medium.

### Information on atherosclerosis patients

The subjects were recruited from outpatients in Kusaka Hospital, Hiroshima, Japan, and those with cancer were excluded. In the present study, all study subjects (83 participants) underwent carotid ultrasonography. The subjects were classified into four levels according to their plaque score as previously described [20,21]. Briefly, the plaque score (mm) is calculated by summing the plaque thicknesses of the three segments (internal carotid, carotid, and common carotid) on each of the right and left sides. Plaque scores of 1 mm or less were defined as normal controls (*n*=21), and plaque scores greater than 1 mm were defined as patients with plaque (*n*=62) [20,21]. Furthermore, patients with plaque were divided into three groups: 1.0–5.0 was mild atherosclerosis, 5.1–10.0 was moderate atherosclerosis, and 10.1 or more was severe atherosclerosis [20,21]. The breakdown of patients with plaque was mild (*n*=28), moderate (*n*=17), and severe (*n*=17). The present study was approved by the Ethics Committee of Hiroshima University, and blood samples were collected after obtaining written informed consent from all participants.

### Quantification of cfDNA in plasma

To compare the levels of n-cfDNA and mt-cfDNA, the absolute copy number of those was measured using qPCR. Peripheral blood was collected in ethylenediaminetetraacetic acid (EDTA) tubes and centrifuged at 3000 rpm for 10 min at 20°C to separate plasma, which was immediately stored at –20°C. For DNA isolation from plasma fractions, the NucleoSpin cfDNA XS (TAKARA, JAPAN) was used with a starting sample volume of 240 µl and an elution volume of 30 µl according to the manufacturer’s protocol. n-cfDNA and mt-cfDNA copy numbers were determined by absolute quantification via real-time qPCR, using an mtDNA primer (NADH1) and nDNA primer (β-globin). For absolute quantification, a five-point standard curve consisting of known copy numbers of mitochondrial and nuclear DNA amplicons (Integrated DNA Technologies, U.S.A.) was applied to each plate in duplicate. The copy number obtained from the standard curve was adjusted to account for the starting and final elution volumes of the sample using the following equation and expressed as copy number/plasma µl [22].

Copy number/μl = copy number × elution volume (μl)/qPCR reaction volume (μl) × sample volume (μl). The sequences are shown in Supplementary Table S1.

### Statistical analysis

Data are expressed as mean ± SEM for continuous variables and frequency (percentage) for categorical variables. Student’s *t*-test or the Mann–Whitney U-test was performed on continuous data, and the Chi-square test was performed on categorical data. In the multivariate logistic regression analysis, variables were included if they met the threshold for statistical significance in the univariate analysis, except smoking (*P*<0.05). Variables included for comparison that met these criteria were age, gender, HDL, eGFR, and cfDNA. Multiple group comparison was analyzed using the Kruskal–Wallis test, followed by the Steel–Dwass test. The correlation between cfDNA copy number and clinical data was analyzed using the Pearson correlation. To investigate if cfDNA (mt-cfDNA and n-cfDNA) was useful as a biomarker for predicting the incidence of atherosclerosis (normal vs plaque), we performed receiver-operating characteristic (ROC) curve analysis using the data of 83 subjects. A *P*-value of less than 0.05 was considered to indicate statistical significance.

## Results

### CSE increased DNA damage in the nucleus and mitochondria of HUVECs

**Figure 1:**
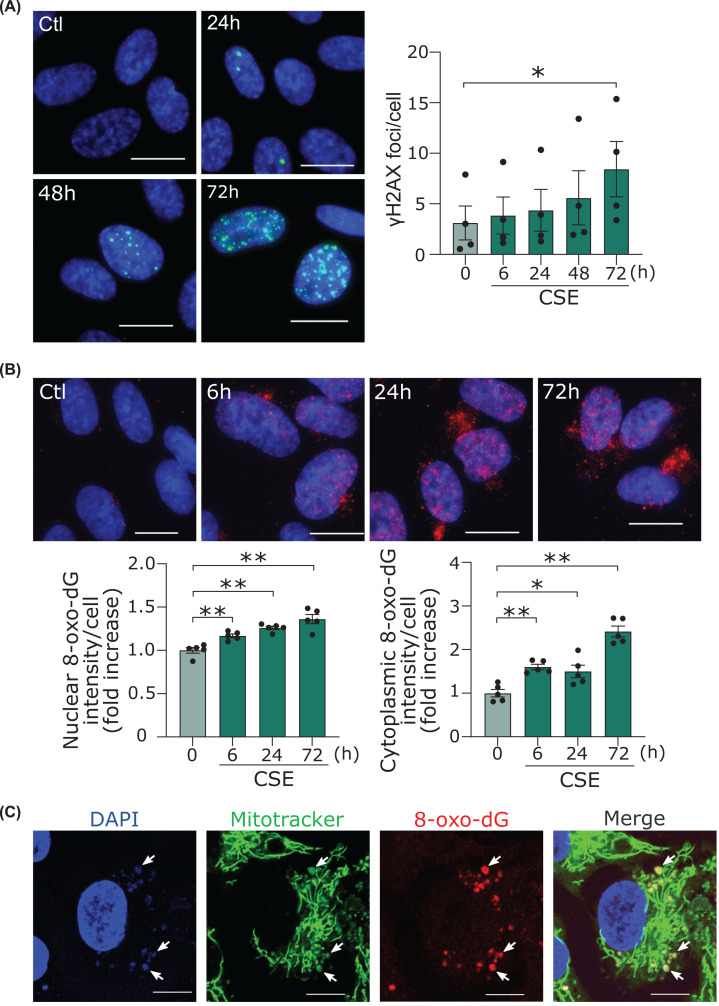
CSE increased nuclear and mitochondrial DNA damage in human endothelial cells (**A**) Immunofluorescent staining of the γH2AX (green) in HUVECs. Scale bar = 20 μm. Time course of γH2AX formation by CSE. *P<0.05 compared with corresponding control (*n*=4). (**B**) Immunofluorescent staining of the 8-OHdG (red) in HUVECs. Scale bar = 20 μm. Time course of the 8-OHdG formation in nuclei and cytoplasm. *P<0.05, **P<0.01 compared with control (*n*=5). (**C**) Images taken by confocal microscopy of immunofluorescent staining of the 8-OHdG (red) and MitoTracker™ RED CMXROS (green) and DAPI (blue) in HUVECs. Scale bar = 20 μm. Because permeabilization was not performed, the 8-OHdG in the nucleus was not stained. Arrows show colocalization of the 8-OHdG in mitochondria. Abbreviations: γH2AX, phosphorylated histone H2AX; 8-OHdG, 8-hydroxy-2′-deoxyguanosine; CSE, cigarette smoke extract.

We first confirmed the type of DNA damage and the time course produced by CSE in HUVECs. Since the initial cellular response upon double-strand break (DSBs) is the phosphorylation of histone H2AX, we performed immunofluorescent staining with antibody against phosphorylated H2AX (γH2AX), the most prominent marker of DSBs [23]. DSBs gradually increased until they became significant 72 h after CSE treatment (Figure 1A; Supplementary Figure S2A). This is relatively slow compared with other DSB-inducing stimuli such as hydrogen peroxide [4] and radiation [24]. CSE also caused oxidative DNA damage assessed by immunostaining with 8-OHdG antibody as early as 6 h and further increased (Figure 1B; Supplementary Figure S2B). Of note, 8-OHdG staining was increased both in nuclei and cytoplasm. Immunostaining using 8-OHdG antibody along with MitoTracker revealed that the increase in the cytoplasm was due to oxidative damge of mtDNA (Figure 1C).

### CSE causes minority mitochondrial outer membrane permeabilization and activation of sublethal apoptotic pathways

**Figure 2:**
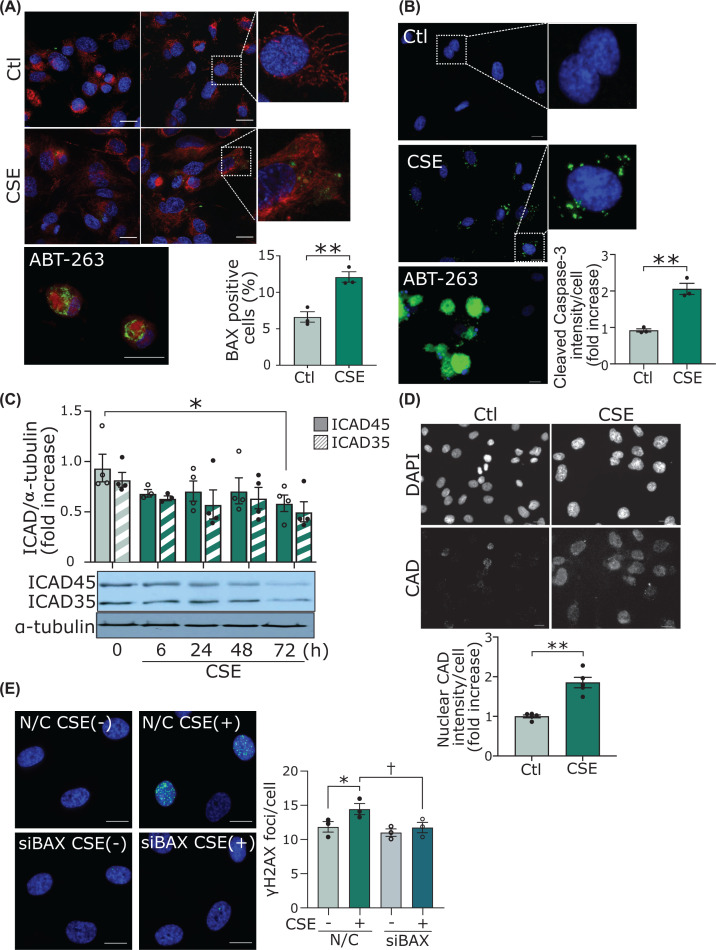
CSE causes minority MOMP and activation of sublethal apoptotic pathways (**A**) Immunofluorescent staining of BAX 6A7 in HUVECs. Scale bar = 20 μm. Cells were treated with CSE for 72 h. As a positive control, cells were treated with ABT-263 for 6 h, which is an inhibitor of Bcl-2. The samples were assessed with an LSM780 confocal laser-scanning microscope (Carl Zeiss, Germany). The percentage of cells with BAX foci were counted. **P<0.01 (*n*=3). (**B**) Immunofluorescent staining of cleaved caspase-3 in HUVECs. Scale bar = 20 μm. Cells were treated with CSE for 72 h. As a positive control, we administered ABT-263 for 6 h. **P<0.01 (*n*=3). (**C**) Time course of cytosolic ICAD45 and ICAD35 levels by Western blot analysis. The bands from Western blot were quantified and standardized to α-tubulin levels. *P<0.05 compared with control of ICAD45 (*n*=4). (**D**) Immunofluorescent staining of CAD in HUVECs. Scale bar = 20 μm. Cells were treated with CSE for 72 h. Quantification of the intensity of CAD in the nucleus. Nuclear regions were analyzed and calculations were based on five different areas of the slide (*n*=5 areas). **P<0.01. (**E**) Immunofluorescent staining of the γH2AX in HUVECs. HUVECs transfected with siRNA against BAX (siBAX), or negative control siRNA (siNC) were treated with CSE for 72 h. *P<0.05 compared with siNC and †*P*<0.05 compared with siNC treated with CSE (*n*=3). Abbreviations: CAD, caspase-activated DNase; ICAD, inhibitor of caspase-activated DNase; siRNA, small-interfering RNA; other abbreviations as in Figure 1.

It has been reported that mitochondrial DNA damage subsequently induces caspase-dependent apoptosis [25], and that the caspase-dependent apoptosis follows proapototic BAK/BAX activation, which causes mitochondrial outer membrane permeabilization (MOMP) [26]. In the present study, CSE decreased the mitochondrial membrane potential in HUVECs (Supplementary Figure S2A) but did not induce apoptosis (Supplementary Figure S2B). This observation prompted us to speculate that CSE partially activates caspase-3 through ‘minority MOMP,’ which is MOMP in only a part of mitochondria [27,28], thereby activating some CAD and resulting in partial DNA fragmentation (i.e., DSBs) [28,29] in nuclei. We investigated the activation of BAX and the downstream signaling. One of the structural changes of BAX activation is the opening of the α1-α2 loop and the exposure of an N-terminal 6A7 epitope [30]. We investigated BAX activation using an antibody that specifically binds to 6A7. As shown in Figure 2A, focal but significant activation of BAX in the mitochondria was observed in the CSE-treated cells, while ABT-263, an inhibitor of Bcl-2, induced massive BAX activation in most mitochondria and appeared to cause apoptosis morphologically. This result indicates that CSE treatment induced MOMP only in part of the mitochondria. In the cells treated with ABT-263, the staining of activated caspase-3 was observed throughout the cells (Figure 2B) and apoptosis was induced (Supplementary Figure S2B). In contrast, CSE treatment significantly but only focally increased the activation of caspase-3 (Figure 2B). CSE reduced the levels of inhibitor of CAD (ICAD)45 and ICAD35, as compared with the control (Figure 2C). Quantification of the Western blot showed that the levels of ICAD45 were significantly decreased at 72 h after CSE addition compared with control. The immunofluorescent study revealed that CAD in the nucleus was significantly increased at 72 h after CSE addition (Figure 2D). Finally, BAX knockdown by siRNA (Supplementary Figure S3A) suppressed the CSE-induced DSBs, suggesting that increased minority MOMP leads to nuclear DSBs (Figure 2E and Supplementary Figure S3B).

### Increase in the level of inflammatory cytokine expression by CSE

**Figure 3:**
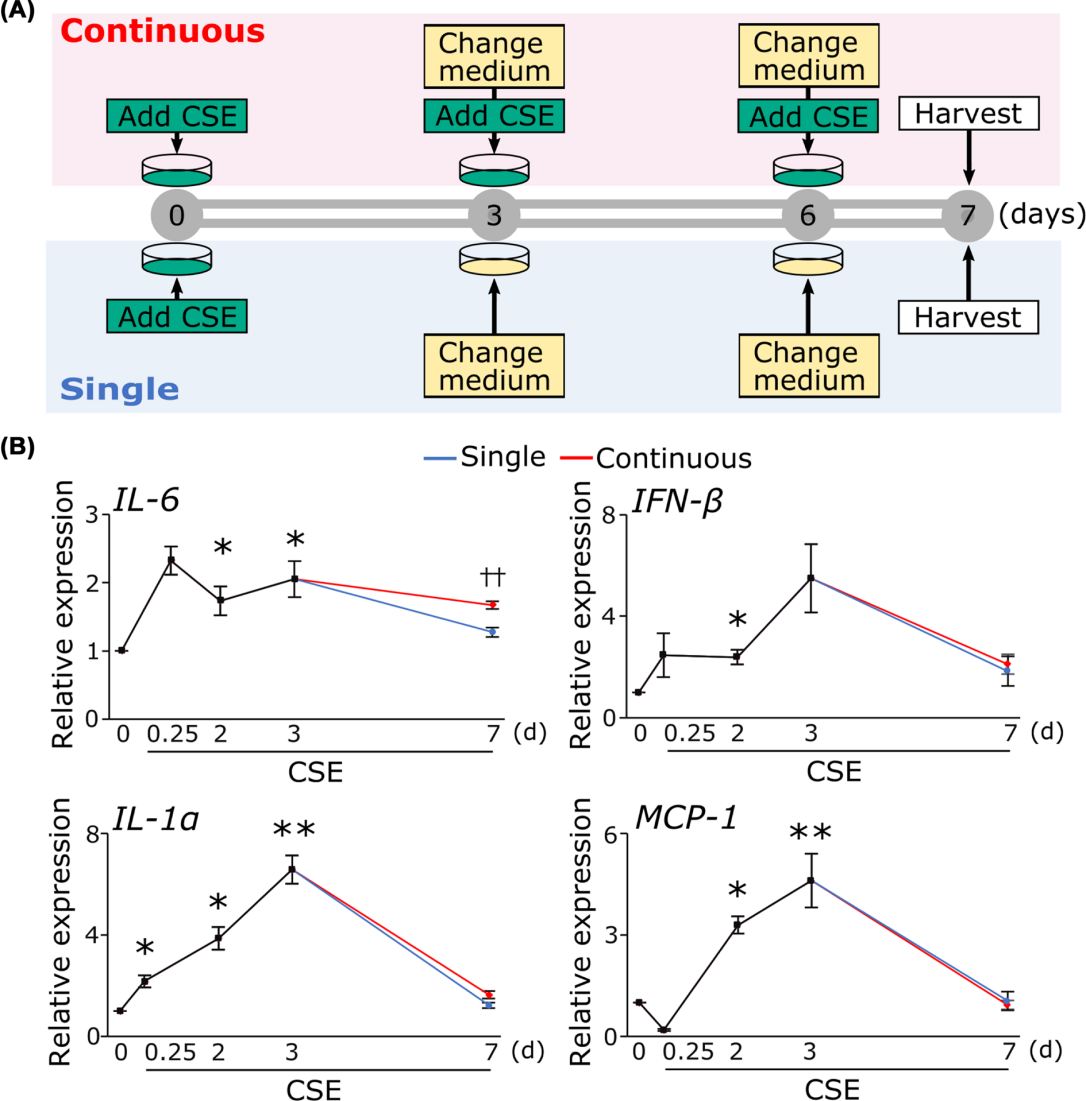
Increase in the mRNA level of inflammatory cytokine expression by CSE (**A**) Time schedule for CSE treatment. (**B**) Cells treated with CSE as described in the text. Quantification of mRNA expression of inflammatory cytokines including IL-6, IL-1α, MCP-1, and IFN-β was performed real-time PCR. *P<0.05, **P<0.01 compared with control (*n*=4). ††*P*<0.01 single vs continuous (*n*=4 or 5). Abbreviations: IFN-β, interferon β; IL-1α, interleukin-1 α; IL-6, interleukin-6; MCP-1, monocyte chemoattractant protein-1; other abbreviations as in Figure 1.

It has been reported that DNA damage causes inflammation [5]. We investigated whether CSE treatment increases inflammatory cytokines. CSE addition increased mRNA expression of L-6, IL-1α, IFN-β, and MCP-1 within 3 days, and they returned to baseline within 7 days (Figure 3B). To examine the expression levels of inflammatory cytokines under sustained exposure to CSE, we replenished HUVECs with CSE every 3 days when replacing the culture medium (Figure 3A), and compared levels of inflammatory cytokines to those in cells stimulated with a single CSE exposure. Cells with continuous CSE exposure maintained increased expression of only IL-6 even after the third day (Figure 3B).

### CSE activates the cGAS-stimulator of interferon genes pathway

**Figure 4:**
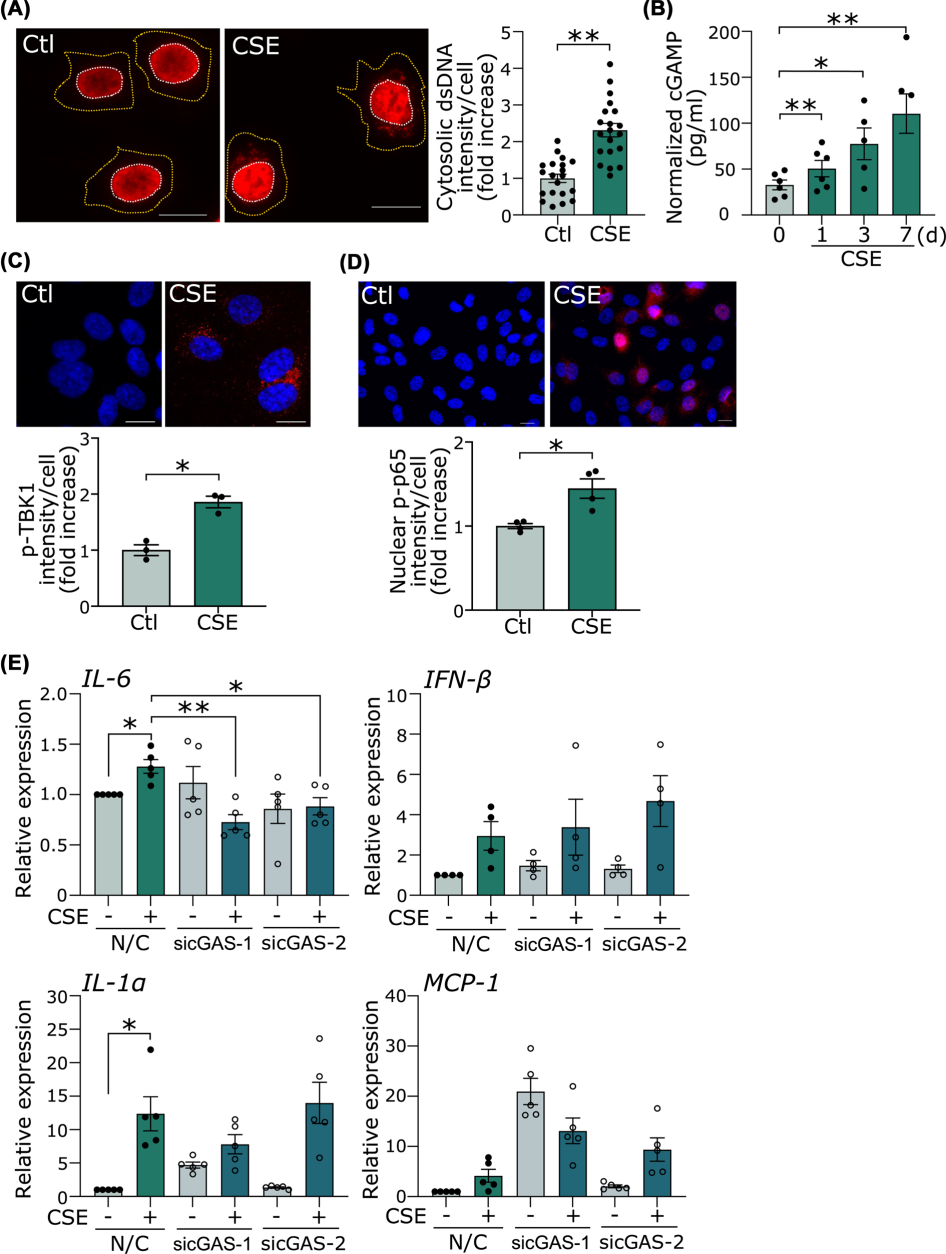
CSE causes the accumulation of cytosolic DNA and activation of cytosolic DNA sensor (**A**) Immunofluorescent staining of dsDNA in HUVECs. Scale bar = 20 μm. Cells were treated with CSE for 24 h. The cell outline (orange dotted line) was determined by observation in bright field. Quantification of the cytosolic dsDNA intensity per cell. **P<0.01 compared with control (*n*=20 cells per group) (**B**) The production of cGAMP was measured by ELISA. The cGAMP levels were normalized by total protein concentration. *P<0.05, **P<0.01 compared with control (*n*=5 or 6). (**C**) Immunofluorescent staining of p-TBK1 in HUVECs. Scale bar = 20 μm. Cells were treated with CSE for 72 h. Quantification of the intensity of pTBK1 in the cytosol. Cytosolic regions were analyzed and calculations were based three different areas of the slide (*n*=3 areas). *P<0.05. (**D**) Immunofluorescent staining of p-p65 in HUVECs. Scale bar = 20 μm. Quantification of the intensity of p-p65 in the nucleus. Nuclear regions were analyzed and calculations were based four different areas of the slide (*n*=4 areas). *P<0.05. (**E**) HUVECs transfected with siRNA against cGAS (sicGAS-1 or sicGAS-2), or siNC were treated with CSE for 7 days. *P<0.05, **P<0.01, (*n*=4 or 5). Abbreviations: dsDNA, double-strand DNA; TBK1, TANK-binding kinase 1; other abbreviations as in Figures 1 and 2.

We investigated whether cytosolic DNA and DNA sensors are involved as a pathway to connect DNA damage to inflammation. The dsDNA antibody was used to verify the increase in cytosolic DNA as a result of CSE-induced DNA damage. The intensity of dsDNA in the cytosol was significantly increased in cells treated with CSE compared with control (Figure 4A). Next, we examined which DNA sensor is involved in CSE-induced inflammation. cGAS and stimulator of interferon genes (STING) are widely expressed in various cells including vascular endothelial cells. The production of cGAMP, the second messenger of cGAS, measured by ELISA, was significantly increased with CSE (Figure 4B). CSE increased the activity of TBK1, a downstream kinase (Figure 4C). NF-κB, one of the downstream molecules, was activated and increased in nuclear localization after CSE treatment, as shown by p-p65 immunostaining (Figure 4D). These results suggest that the cGAS-STING-NF-κB axis was activated by CSE. We then examined whether the sustained increase in IL-6 mRNA expression by CSE was dependent on the activation of the cGAS-STING pathway. HUVECs were transfected either with siRNA against cGAS (sicGAS-1 or sicGAS-2) or siNC (Supplementary Figure S4). The increase in IL-6 mRNA expression on the seventh day after CSE stimulation was significantly suppressed by sicGAS-1 and sicGAS-2, while the mRNA expression of IL-1α, MCP-1, and IFN-β was not (Figure 4E). These results suggest that the cGAS-STING pathway was involved in the sustained increase in IL-6 mRNA expression. On the other hand, CSE did not induce caspase-1 activation, as demonstrated by the generation of cleaved caspase-1, suggesting that inflammasome is not involved in the CSE-induced expression of cytokines (Supplementary Figure S5). We could not detect significant TLR9 expression in HUVECs with quantitative RT-PCR (data not shown).

### CSE causes accumulation of both nDNA and mtDNA in the cytosol

**Figure 5:**
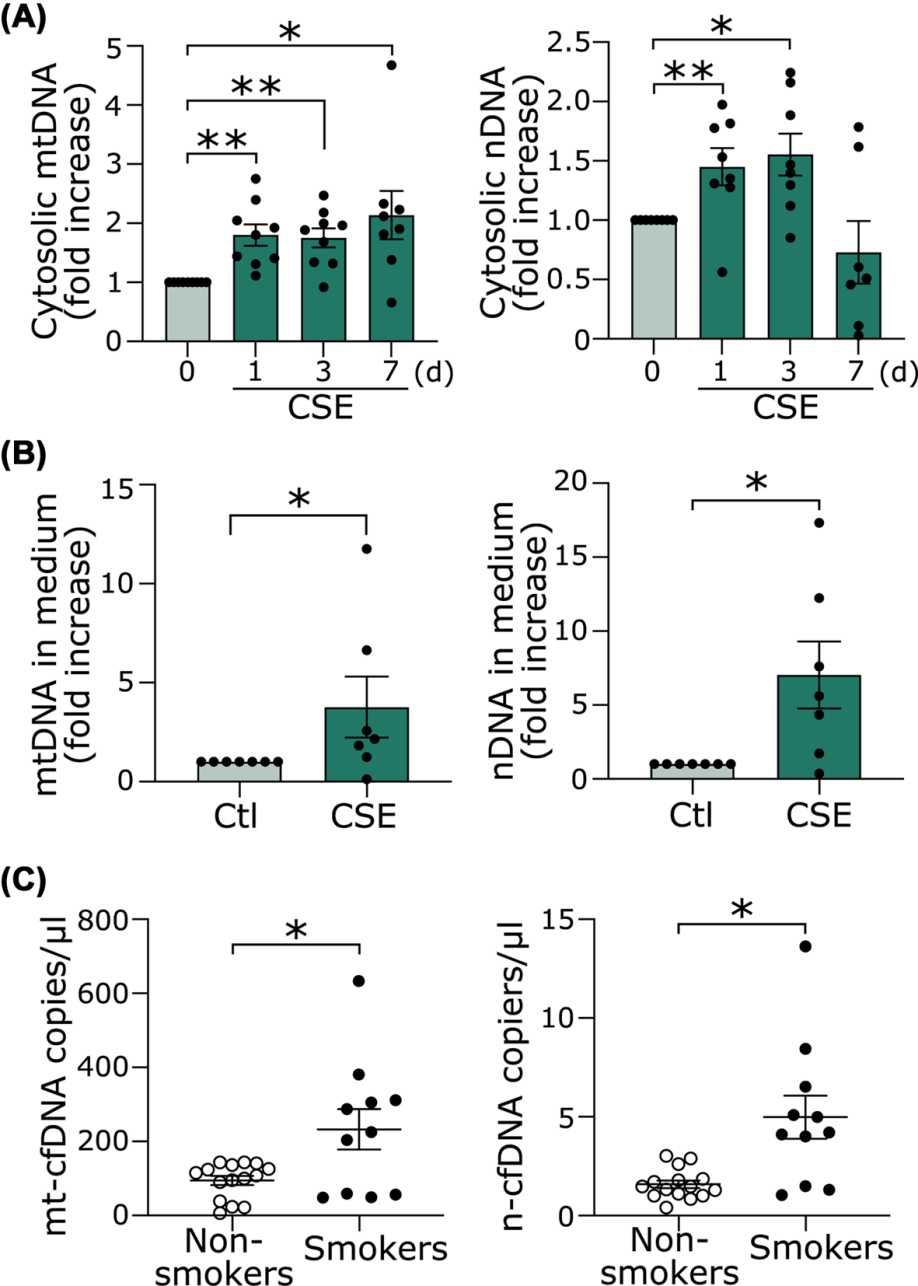
CSE causes accumulation of both nDNA and mtDNA in the cytosol and extracellular space (**A**) Cytosolic nDNA and mtDNA were quantitated via qPCR using nDNA primers (β-globin) or mtDNA primers (NADH1). *P<0.05, **P<0.01 compared with control (*n*=7 or 9). (**B**) Cells were treated with CSE for 48 h. n-cfDNA and mt-cfDNA in the medium were quantitated. *P<0.05 (*n*=6). (**C**) n-cfDNA and mt-cfDNA in the plasma of smokers (*n*=11) and nonsmokers (*n*=15) were quantitated. *P<0.05.

When MOMP occurs, mtDNA may be released with other mitochondria contents through BAK/BAX [31]. As shown above, the minority MOMP by CSE did not induce apoptosis, and thus mtDNA may accumulate in the cytosol. In addition, nuclear DSBs and oxidative DNA damage may also cause accumulation of cytosolic DNA. Thus, we determined whether the increased cytosolic dsDNA shown in Figure 4A was derived from nuclei or mitochondria with real-time PCR by using the specific primers for the nucleus and mitochondria. Both mtDNA and nDNA were significantly increased after 1 day of CSE treatment (Figure 5A).

### CSE increases cfDNA

It has been reported that DNA accumulated in the cytosol is released outside the cell as cfDNA, although the detailed mechanism is unclear [7,8]. As shown in Figure 5B, both mt-cfDNA and n-cfDNA were increased in the conditioned media from HUVECs treated by CSE. Based on these *in vitro* results, we investigated whether cfDNA is increased in the plasma of young healthy smokers. The subject characteristics are shown in Table 1. There were no significant differences in age, gender, or body mass index (BMI) between the smokers and nonsmokers. Both mt-cfDNA and n-cfDNA were increased in the smokers compared with the nonsmokers (Figure 5C).

**Table 1:** Baseline characteristics of the smokers and nonsmokers

	Nonsmoker *n*=15	Smoker *n*=11	*P*-value
Age	35 (26.5–36.5)	36 (29.5–37.5)	0.406
Male, n (%)	11 (73.3%)	11 (100%)	0.063
BMI	22 (19.7–23.1)	19.8 (18.4–21.2)	0.139
Pack years	0	8.25 (4.1–12.8)	<0.001

Continuous data were expressed as median and interquartile range (IQR), and categorical data as number and ratio. Mann–Whitney U test was performed for continuous data, and the Chi-square test was for categorical data.

Abbreviations: BMI, body mass index; Pack year, calculated by multiplying the number of packs of cigarettes smoked per day by the number of years.

### Elevation of cfDNA in atherosclerotic patients

**Figure 6:**
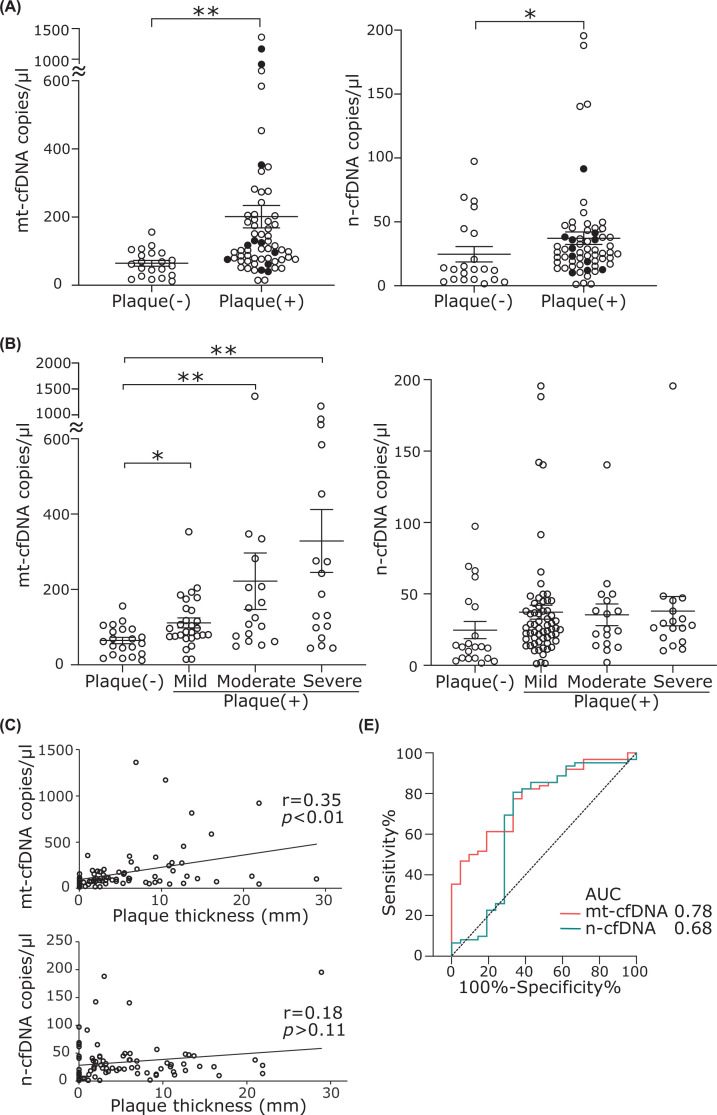
The cf-DNA of atherosclerosis patients (**A**) The cfDNA copy number in normal subject (Plaque [-]) and subjects with carotid plaques. *P<0.05, **P<0.01 compared with Plaque (-). Closed circles represent current smokers. (**B**) Comparison of mt-cfDNA and n-cfDNA among four groups. *P<0.05, **P<0.01 compared with Plaque (-). (**C**) Correlation of cfDNA copy number with plaque thickness. (**D**) ROC analysis for atherosclerosis incidence (plaque [-] vs. plaque[+]), using mt-cfDNA and n-cfDNA. Abbreviations: AUC, area under the curve; mt-cfDNA, mitochondrial cell-free DNA; n-cfDNA, nuclear cell-free DNA.

We have previously reported that DNA damage absent from normal arteries is found at sites of atherosclerosis [4]. Based on the results of our experiments with HUVECs, we hypothesized that cfDNA is increased in the plasma of patients with atherosclerosis. The cfDNA was measured in 83 subjects who visited the hospital for medical checkups or the treatment of lifestyle-related diseases. All study subjects underwent carotid ultrasonography. The subjects were divided into two groups: 21 normal controls and 62 patients with plaques. The median age (IQR) was 74 (67–80) years and 49.3% were male. Most of the subjects had at least one underlying comorbidity; hypertension (*n*=72%), diabetes mellitus (*n*=50%), and dyslipidemia (*n*=53%) were the most common comorbidities (Table 2). The patients with plaques had higher blood levels of both mt-cfDNA and n-cfDNA than the normal controls (Figure 6A). The copy numbers of cfDNA in current smokers (closed circle in Figure 6A) were comparable with those in nonsmokers, suggesting that the effects of smoking are more evident at a younger age. In the current study population, we also performed an analysis that included only nonsmokers (Supplementary Figure S6). As in the overall analysis, the analysis of nonsmokers showed a significant increase in plasma cfDNA in subjects with plaques compared with those without plaques (Supplementary Figure S6). Next, we performed a multivariate analysis to investigate whether cfDNA is an independent risk factor for carotid atherosclerotic plaque. Variables that had statistical significance in the univariate analysis were included. Multivariate analysis showed that mt-cfDNA is an independent risk factor for plaque (OR = 3.93, 95% CI: 1.31–11.7, *P*=0.005; Table 3). Multivariate analysis in nonsmokers also show that mt-cfDNA is an independent risk factor for plaque (OR = 3.83, 95% CI: 1.39–13.38, *P*=0.007; Supplementary Table S3). The degree of carotid atherosclerosis was classified into four groups based on the severity of the plaques, as described in the methods section. The subject characteristics are shown in Supplementary Table S4. The amount of mt-cfDNA was significantly increased in all plaque groups compared with the normal controls, but there was no significant difference in n-cfDNA among the groups (Figure 6B). Furthermore, the thickness of plaques correlated with copy numbers of mt-cfDNA but not with those of n-cfDNA (Figure 6C). Finally, we performed ROC curve analysis to determine the diagnostic utility of mt-cfDNA and n-cfDNA for predicting the incidence of atherosclerosis (Figure 6D). The area under the curve (AUC) was 0.78 (95% CI: 0.67–0.88) for mt-cfDNA, and 0.68 (95% CI: 0.52–0.83) for n-cfDNA.

**Table 2:** Baseline characteristics of study subjects

	Total *n*=83	Plaque (-) *n*=21	Plaque (+) *n*=62	*P*-value
Age	74 (66.5–79.8)	64.5 (55.8–72.5)	75 (70–81)	<0.0001
Male, n (%)	41 (49.3%)	6 (28.6%)	35 (56.5%)	0.014
Smoking, n (%)	11 (13.4%)	0 (0%)	11 (17.7%)	0.043
HbA1c	6.2 (5.6–6.7)	5.9 (5.5–6.6)	6.2 (5.7–6.7)	0.93
TG	97 (69–120)	76 (61–103)	101 (74–125)	0.062
HDL-C	61 (51–71)	71 (63–77)	58 (49–66)	0.006
LDL-C	103 (83.8–125)	113 (95–142)	101 (82–122)	0.49
AST	22 (19–26)	21 (19–24)	22.5 (19–27)	0.51
ALT	17 (14–24)	16 (14–27)	18 (13–23)	0.53
eGFR	59.6 (48–71)	70.4 (62.2–73.5)	55.3 (45.9–69.1)	0.028
MBP	92.5 (83.8–99.6)	96 (81.6–102)	92.3 (85.3–98.9)	0.84
BMI	24.7 (21.4–27.1)	23 (20.8–26.6)	25 (22–27)	0.14
Hypertension, n (%)	60 (72%)	13 (62%)	47 (75.8%)	0.34
Diabetes, n (%)	42 (50.6%)	9 (42.9)	33 (53.2%)	0.8
Dyslipidemia, n (%)	44 (53%)	10 (47.6%)	34 (54.8%)	0.44
Atrial fibrillation, n (%)	4 (4.8%)	0	4 (6.5%)	0.32
Heart failure, n (%)	1 (1.2%)	0	1 (1.6%)	0.42

Continuous data were expressed as median and IQR, and categorical data as number and ratio. Mann–Whitney U test was performed for continuous data, and the Chi-square test was for categorical data.

Abbreviations: ALT, alanine aminotransferase; AST, aspartate transaminase; BMI, body mass index; eGFR, estimated glomerular filtration rate; HbA1c, hemoglobin A1c; HDL-C, high-density lipoprotein cholesterol; LDL-C, low-density lipoprotein cholesterol; MBP, mean blood pressure; TG, triglyceride.

**Table 3:** Logistic regression analysis: associations between the presence of plaque and the log cfDNA and clinical profiles

	Univariate			Multivariate		
	OR	95% CI	*P*-value	OR	95% CI	*P-*value
log mt-cfDNA	3.89	1.83–9.88	<0.001	3.93	1.46–13.49	0.005
log n-cfDNA	1.80	1.10–3.09	0.02	1.10	0.59–2.09	0.76
Age	1.10	1.04–1.17	<0.001	1.06	0.99–1.14	0.07
Male	3.89	1.32–13.19	0.01	1.45	0.35–6.26	0.61
BMI	1.02	0.92–1.13	0.72			
Hypertension	1.69	0.55–4.95	0.35			
Dyslipidemia	1.48	0.54–4.17	0.44			
Diabetes	1.14	0.41–3.15	0.80			
TG	1.01	0.99–1.02	0.14			
HDL-C	0.95	0.92–0.99	0.007	0.98	0.94–1.03	0.39
LDL-C	0.99	0.98–1.001	0.11			
HbA1c	0.96	0.68–1.01	0.11			
eGFR	0.96	0.93–0.99	0.008	0.97	0.93–1.02	0.25

Multivariate analysis was performed with the presence of plaque as binary dependent variables and with the log cfDNA, age, male, HDL-C, eGFR as covariates.

Abbreviations: BMI, body mass index; eGFR, estimated glomerular filtration rate; HbA1c, Hemoglobin A1c; HDL-C, high-density lipoprotein cholesterol; LDL-C, low-density lipoprotein cholesterol; mt-cfDNA, mitochondrial cell-free DNA; n-cfDNA, nuclear cell-free DNA; TG, Triglyceride.

## Discussion

The major findings of this study were: (1) continuous exposure to CSE induces not only nuclear but also mtDNA damage, which leads to cytosolic DNA accumulation, and evokes chronic inflammation via the cGAS-STING pathway in endothelial cells; and (2) free DNA in the cytosol is transferred to the extracellular space *in vitro* and the cfDNA, especially mt-cfDNA in the blood of patients with carotid atherosclerosis was significantly increased compared with the normal controls and was correlated with severity. Thus, mt-cfDNA may become a useful biomarker for atherosclerosis screening, reflecting biological condition as well as genomic and mtDNA integrity in the vascular endothelium. To the best of our knowledge, this is the first paper to show an increase in mt-cfDNA in human atherosclerosis. The use of mt-cfDNA has the potential to screen for atherosclerosis using a blood sample alone, without the use of special equipment or procedures, such as carotid artery echocardiography.

Cigarette smoke contains not only nicotine and tar but also thousands of other chemicals. To investigate the effects of smoking on endothelial cells, we used CSE prepared from the water-soluble components of cigarette smoke. The CSE concentration added to the cells in the present study is equivalent to 12.3 cigarettes for a human with a blood volume of 4.6 l, which corresponds to a realistic amount of human daily exposure to cigarette smoke.

Given that the generation of DSBs by the CSE took 72 h, CSE-induced DSBs do not seem to be formed directly by CSE or to be caused by reactive oxygen species but by the overall effect of cigarette smoke because the time course is different from and much slower than those generated by reactive oxygen species [4]. In the present study, we showed that CSE induced partial fragmentation of nuclear DNA by inducing partial BAX activation, i.e., a minority MOMP [28], and caspase-3 activation, followed by caspase 3-mediated ICAD cleavage and nuclear translocation of CAD. In addition, silencing BAX suppressed the CSE-induced nuclear DSBs. These data suggest that CSE-induced nuclear DSBs occurred via minority MOMP. Surprisingly, oxidative DNA damage occurred not only in the nucleus but also in mitochondria. Recently, Li et al. reported that electronic cigarettes induced mitochondrial DNA damage [32], although the type of DNA damage has not been investigated. The predominant repair pathway for oxidative damage in mitochondoria is base excision repair (BER) [33]. BER removes small lesions such as oxidized bases. Thus, we speculate that oxidative damage of mtDNA and its repair, and the activation of BAK/BAX [31] is the mechanism by which mtDNA is released to cytosol.

We found that CSE-induced DNA damage resulted in the accumulation of nDNA and mtDNA in the cytosol. Mammalian cells originally have a pathway that detects RNA and DNA viruses, and triggers inflammation as a defense mechanism. It has been reported that the sensors can be activated not only by foreign DNA but also by self-DNA [5]. Furthermore, it was recently reported that this sensor can be activated by mtDNA as well as nDNA [34,35]. Our study showed that CSE treatment increased the production of cGAMP and phosphorylation of TBK1, strongly suggesting the involvement of the cGAS-STING pathway. In our study, inflammatory cytokine downstream of the cGAS-STING pathway was IL-6, but not IFN-β. This result is similar to those from a study by Maekawa et al., who reported that cisplatin increased cytosolic mtDNA, which activated the cGAS-STING pathway, thereby increasing IL-6 expression in primary cultured tubular cells [16]. In contrast, Takahashi et al. reported that cytoplasmic accumulation of nuclear DNA in senescent cells play key roles to induce SASP, especially IFN-β, via activation of the cGAS-STING pathway in fibroblasts [36]. We speculate that the downstream signaling of cGAS-STING is dependent on the cell type or stimulus. Toll-like receptor 9 (TLR9) is one of the DNA sensors [37]. Li et al. reported that electronic cigarettes elevated mtDNA levels in circulating blood and induced the expression of TLR9, which elevated the expression of proinflammatory cytokines in macrophages and consequently led to atherosclerosis in ApoE knockout mice [32]. However, in the present study, TLR9 was rarely expressed in HUVECs, and TLR-9 knockdown could not decrease the expression of IL-6 that was consistent with the results of another study [37] (data not shown). DNA recognition by sensors is known to depend on the length, modifications, motifs, subcellular localization, and structure of the DNA [38]. In addition, the specific cytosolic sensors that lead to inflammation may differ depending on the cell types or the origins of the DNA [38].

The cfDNA is widely studied as a disease biomarker in the clinical setting [9]. We measured cfDNA in plasma, but some studies measured cfDNA in serum. It has been reported that serum contains significantly more cfDNA than plasma [22]. The serum could have shown a significantly higher concentration of cfDNA due to the release of cellular DNA during the blood clotting procedures, which may mask the original pathophysiological conditions of the patients [39]. Thus, we measured cfDNA in plasma to determine the significance of cfDNA in atherosclerotic patients *in vivo*.

The cfDNA of young healthy smokers was increased compared with that of age-matched nonsmokers. Given the results of the *in vitro* experiments in the present study, which showed that CSE caused nuclear and mtDNA damage in vascular endothelial cells as well as the release of DNA into the medium, it is likely that smoking induces inflammation through cytosolic DNA sensors, and that the continuous release of cfDNA from endothelial cells further exacerbates inflammation through the involvement of surrounding inflammatory cells [32], thereby resulting in the development of atherosclerosis. As expected, there was an increase in cfDNA in the patients with atherosclerosis compared with that of the normal controls. In the present study, only a small percentage of atherosclerosis patients were smokers, suggesting that not only smoking but also other combined risk factors may damage cell nuclei and mitochondria [40–42], leading to inflammation and atherosclerosis. We found that both mt-cfDNA and n-cfDNA were increased in the patients with atherosclerosis, and our multivariate analysis revealed that only mt-cfDNA copy number predicted the risk of atherosclerosis. The mt-cfDNA and n-cfDNA have been reported to show increases in various diseases. Andargie et al. reported that the amount of n-cfDNA on the day of ICU admission in patients of COVID-19 who ultimately died was increased compared with those who survived, but there was no significant difference in mt-cfDNA levels [43]. When cfDNA was measured during severe trauma, trauma patients who subsequently developed chronic severe disease had significantly elevated n-cfDNA, but not mt-cfDNA levels, compared with trauma patients who had rapid clinical recovery [44]. Based on our findings and those of other reports, mt-cfDNA may be caused by chronic cellular damage, while n-cfDNA may be caused by acute cell death. Stortz et al. also mentioned the possibility that n-cfDNA is released only upon cell death, whereas mt-cfDNA is released not only upon cell death but also by active secretory processes [44].

There were some limitations in the study of cfDNA measurement in atherosclerotic patients. First, age was significantly different between the patients with atherosclerosis and the normal controls. Plaque is mostly present in older people, and there were few older people without plaque. In the present study, no correlation was found between age and either n-cfDNA or mt-cfDNA levels (Supplementary Figure S7). Additionally, multivariate analysis showed that age was not an independent risk factor in our study cohort. Thus, it is unlikely that age was a confounding factor. Second, the subjects of the present study were patients with relatively mild atherosclerosis who did not have a history of cardiovascular events. In the future, it will be necessary to examine whether mit-cfDNA is useful in determining advanced cardiovascular disease, such as coronary artery disease. Otherwise, mt-cfDNA may be a marker that can detect patients with early atherosclerosis and help prevent the development of cardiovascular disease by early intervention. Third, the present study is a cross-sectional one with a small number of subjects. A prospective study with a larger population is needed to study whether plasma mt-cfDNA is truly capable of predicting the onset and progression of atherosclerosis [45].

In recent years, electronic cigarette use has risen rapidly. In particular, electronic cigarettes have been marketed to young adults using a variety of flavors. One study showed that low concentrations of selected flavors (vanillin, menthol, cinnamaldehyde, eugenol, and acetylpyridine) induced both inflammation and impaired A23187-stimulated nitric oxide production in endothelial cells, effects that may contribute to cardiovascular toxicity [46]. Thus, in the future, it will be valuable to investigate whether electronic cigarettes and their specific flavors have any effect on mtDNA. Most importantly, although it has been reported that there is increased mtDNA damage in human atherosclerotic plaques and that this damage is associated with high-risk lesions [45], it remains to be determined what factors besides smoking trigger mtDNA damage and the release of mt-cfDNA in the context of atherosclerosis, and what mechanisms are involved.

## Conclusion

The data presented herein identify a novel biomarker for atherosclerosis based on *in vitro* and *in vivo* experiments. In summary, continuous exposure to CSE induces oxidative DNA damage in the nucleus and mitochondria. Mitochondrial damage by CSE is followed by minority MOMP, leading to nuclear DNA fragmentation, i.e., DSBs, both of which cause cytosolic DNA accumulation, and evoke chronic inflammation via the cGAS-STING pathway. Damaged DNA is also released into the extracellular space. mt-cfDNA and n-cfDNA were increased in young smokers and patients with atherosclerosis, but the copy number of mt-cfDNA was more strongly associated with atherosclerosis than that of n-cfDNA. Our study suggests mt-cfDNA in blood as a promising new biomarker for atherosclerosis that reflects biological condition and genomic and mtDNA integrity in the vascular cells.

## Clinical perspectives

- The molecular mechanisms whereby smoking-related DNA damage causes atherosclerosis remain unclear.
- The continuous exposure to CSE induces not only nuclear but also mtDNA damage, which leads to cytosolic DNA accumulation, and evokes chronic inflammation via the cGAS-STING pathway. mt-cfDNA and n-cfDNA in plasma were increased in young healthy smokers and atherosclerosis patients.
- mt-cfDNA in plasma may be a promising new biomarker reflecting the biological and DNA integrity in the vascular cells, and can predict the risk of atherosclerosis.

## Supplementary Material

Supplementary Figures S1-S7 and Tables S1-S4

## Data Availability

Data presented in this manuscript are available from the corresponding authors upon reasonable requests.

## Abbreviations

γH2AX: phosphorylated histone H2AX
8-OHdG: 8-hydroxy-2′-deoxyguanosine
AUC: area under the curve
BER: base excision repair
BMI: body mass index
CAD: caspase-activated DNase
cfDNA: cell-free DNA
cGAS: cyclic GMP-AMP synthase
CSE: cigarette smoke extract
Ct: cycle threshold
DAPI: 4’,6-diamidino-2-phenylindole
DSB: double-strand break
eGFR: estimated glomerular filtration rate
HASM: human aortic smooth muscle cell
HDL: high-density lipoprotein
HUVEC: human umbilical vein endothelial cell
ICAD: inhibitor of caspase-activated DNase
IFN-β: interferon-β
IL-1α: interleukin 1α
IL-6: interleukin 6
IQR: interquartile range
MCP-1: monocyte chemoattractant molecule 1
MOMP: mitochondrial outer membrane permeabilization
mt-cfDNA: mitochondrial cfDNA
mtDNA: mitochondrial DNA
NADH1: NADH dehydrogenase 1
n-cfDNA: nuclear cfDNA
nDNA: nuclear DNA
OR: odds ratio
p-TBK1: phospho-TANK binding kinase 1
siNC: negative control siRNA
ROC: receiver-operating characteristic
SASP: senescence-associated secretory phenotype
STING: stimulator of interferon genes
TLR9: Toll-like receptor 9
