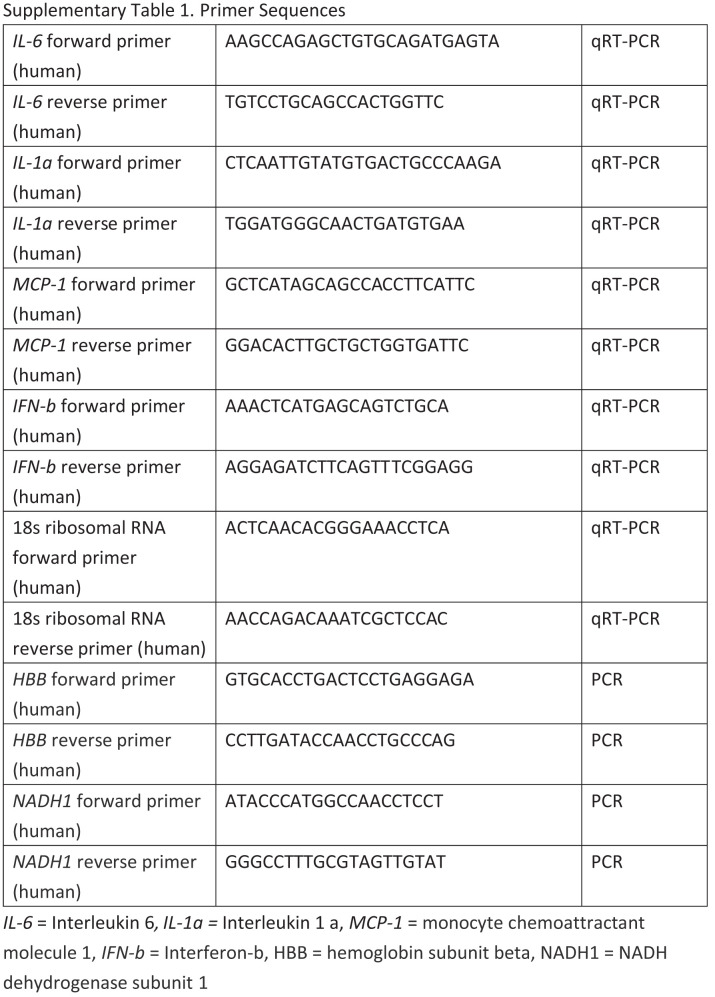# Correction: Cigarette smoke induces mitochondrial DNA damage and activates cGAS-STING pathway -Application to a biomarker for atherosclerosis

**DOI:** 10.1042/CS-2022-0525_COR

**Published:** 2023-03-01

**Keywords:** biomarker, cell-free DNA, cGAS-STING, DNA damage, mitochondria

The authors of the original article would like to correct Supplementary Table 1 of their paper. In the revised Supplementary Table 1 presented here, the primer sequences of HBB and NADH1 in the bottom four rows of Supplementary Table1 have been corrected.