# Supplementary material for: Cigarette smoke induces mitochondrial DNA damage and activates cGAS-STING pathway: application to a biomarker for atherosclerosis
Source: Clin Sci (Lond). 2023 Jan 24;137(2):163–80. doi: 10.1042/CS20220525 (PMC9874975; doi:10.1042/CS20220525)
Supplement: Supplementary Figures S1-S7 and Tables S1-S4 [file CS-2022-0525_supp.pdf]

SUPPLEMENTARY DATA (Supplementary Figures and Tables)

Cigarette smoke induces mitochondrial DNA damage and activates cGAS-STING pathway -Application to a biomarker for atherosclerosis

Keitaro Ueda[1], Chiemi Sakai[1], Takafumi Ishida[2], Kosuke Morita[1], Yusuke Kobayashi[3], Yasunori Horikoshi[4], Akiko Baba[1], Yuma Okazaki[1], Masao Yoshizumi[1], Satoshi Tashiro[4], Mari Ishida[1]

1. Department of Cardiovascular Physiology and Medicine, Graduate School of Biomedical and Health Sciences, Hiroshima University, Hiroshima 734-8551, Japan.
2. Department of Cardiovascular Medicine, Fukushima Medical University, Fukushima 960-1295 Japan.
3. Department of Cardiovascular Medicine, Graduate School of Biomedical and Health Sciences, Hiroshima University, Hiroshima 734-8551, Japan.
4. Research Institute for Radiation Biology and Medicine, Hiroshima University, Hiroshima 734-8551, Japan.

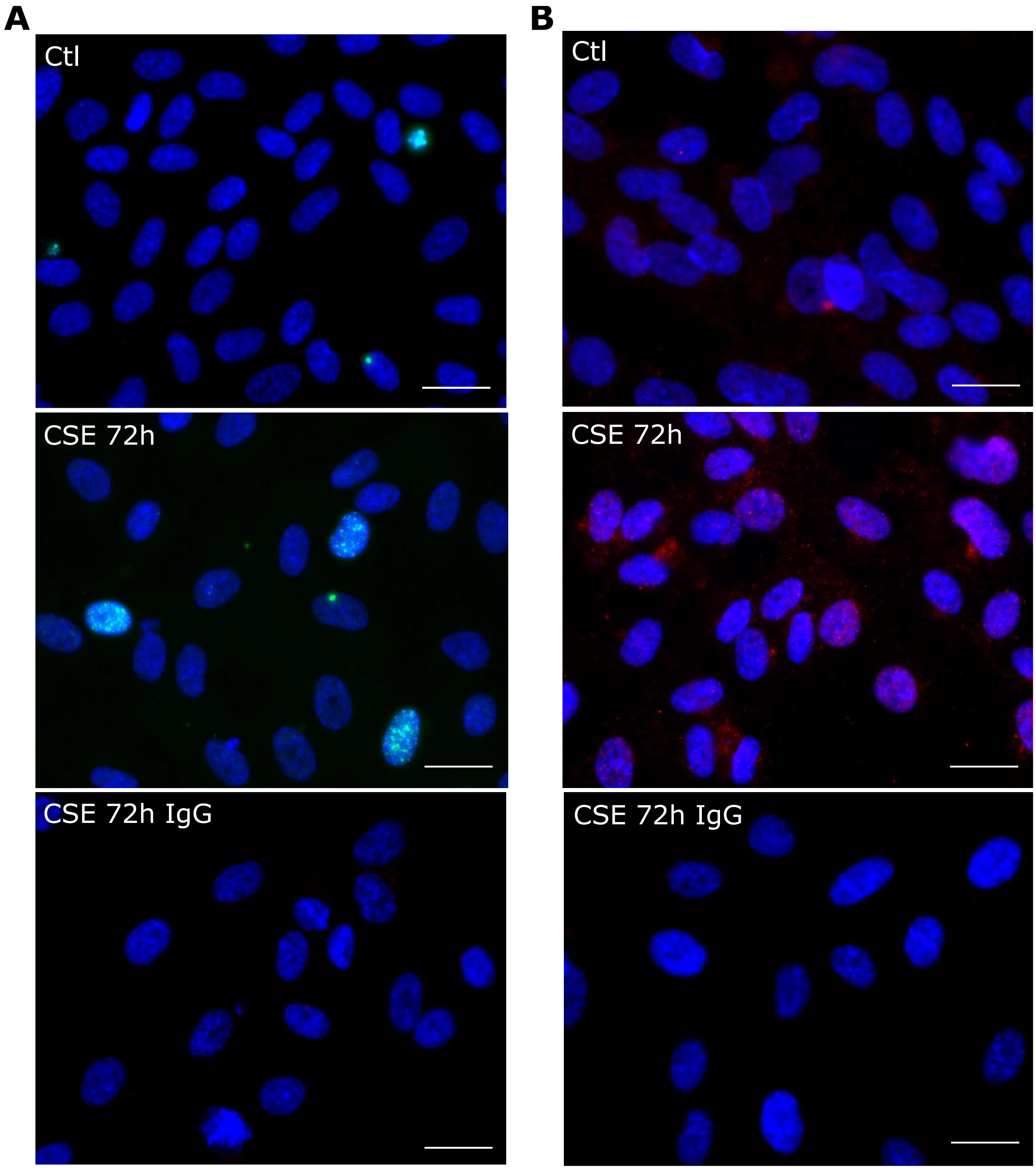
A
Ctl
CSE 72h
CSE 72h IgG
B
Ctl
CSE 72h
CSE 72h IgG

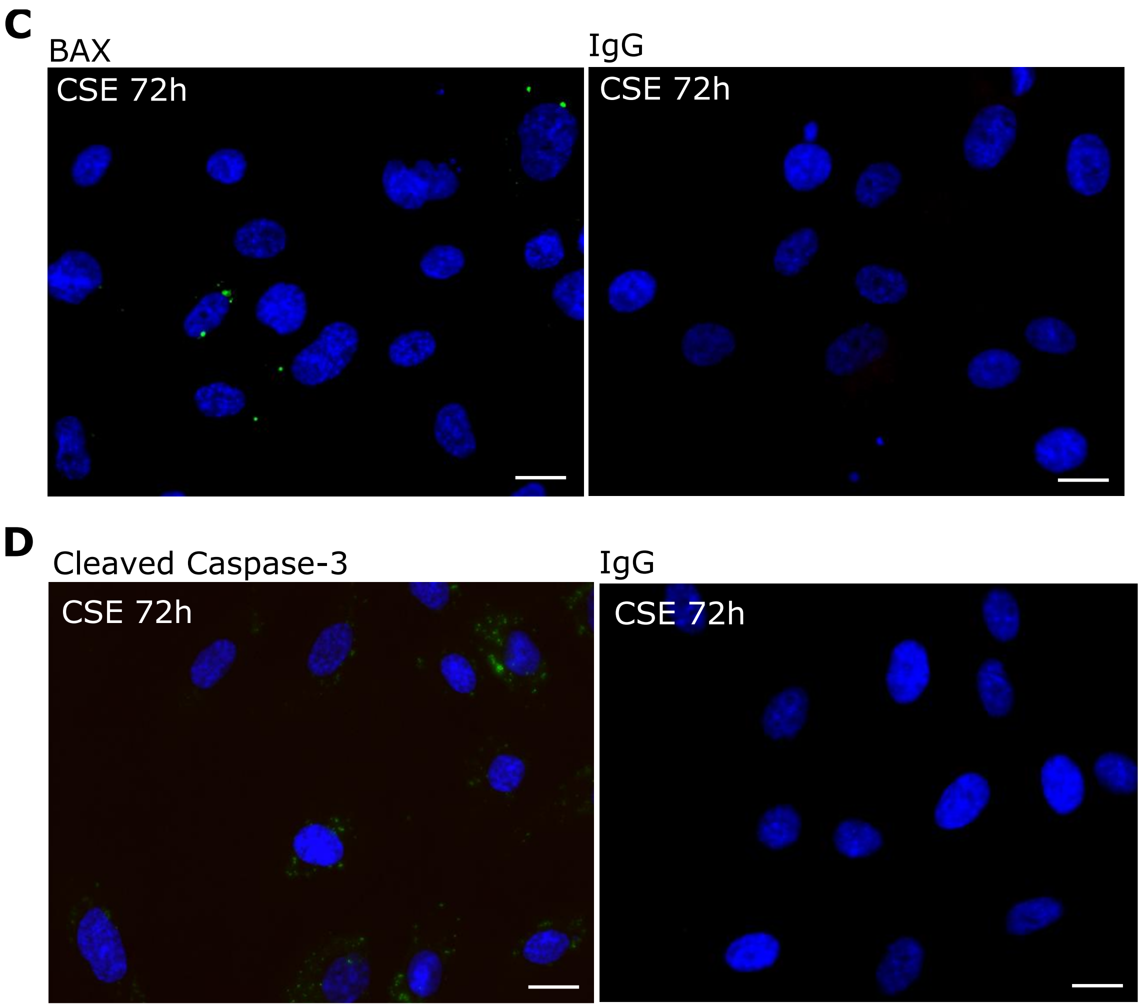

Supplementary Figure 1

(A) Low-power field images of γH2AX immunofluorescent staining (green) in human umbilical vein endothelial cells (HUVECs). Control experiments were performed with normal mouse IgG. γH2AX formation by cigarette smoke extract (CSE) were shown. (B) Low-power field images of 8-OHdG immunofluorescent staining (red) in HUVECs. Control experiments were performed with normal rabbit IgG. (C) Immunofluorescent staining of BAX 6A7 in HUVECs. Control experiments were performed with normal mouse IgG. (D) Immunofluorescent staining of cleaved caspase-3 in HUVECs. Control experiments were performed with normal rabbit IgG.

Scale bar = 50 μm.

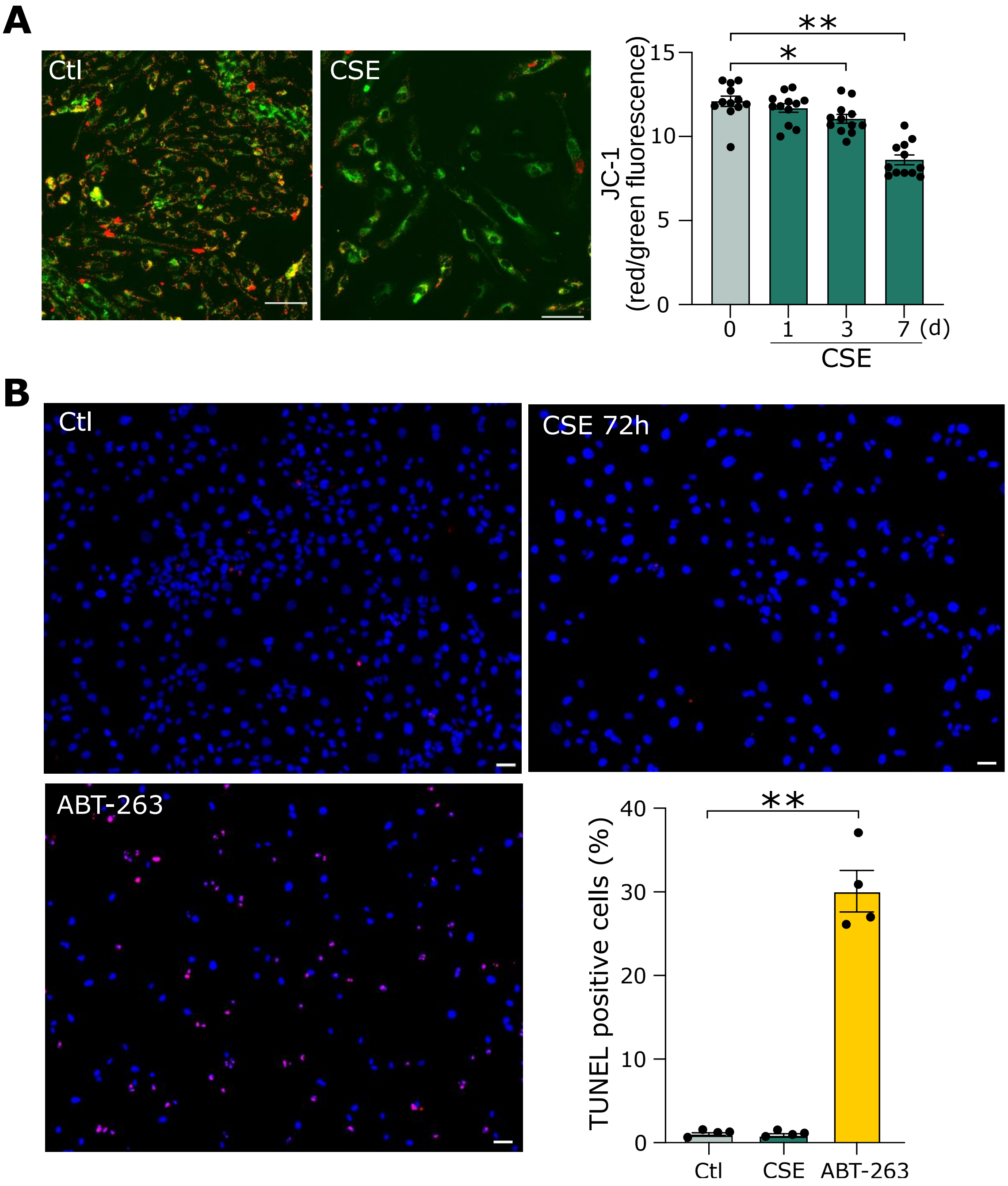

Supplementary Figure 2

(A) Mitochondrial membrane potential after CSE treatment. Scale bar = 100 μm. Mitochondrial membrane potential was quantified by JC-1 probe over seven days with CSE in HUVECs. The ratio of red/green fluorescence indicates mitochondrial transmembrane potential. *P < 0.05, **P < 0.01 compared with control (0 d). (B) TUNEL staining in HUVECs treated with CSE for 72 hours. ABT-263, an inhibitor of Bcl-2 was used for a positive control. Scale bar = 50 μm. **P < 0.01 compared with control (Ctl).

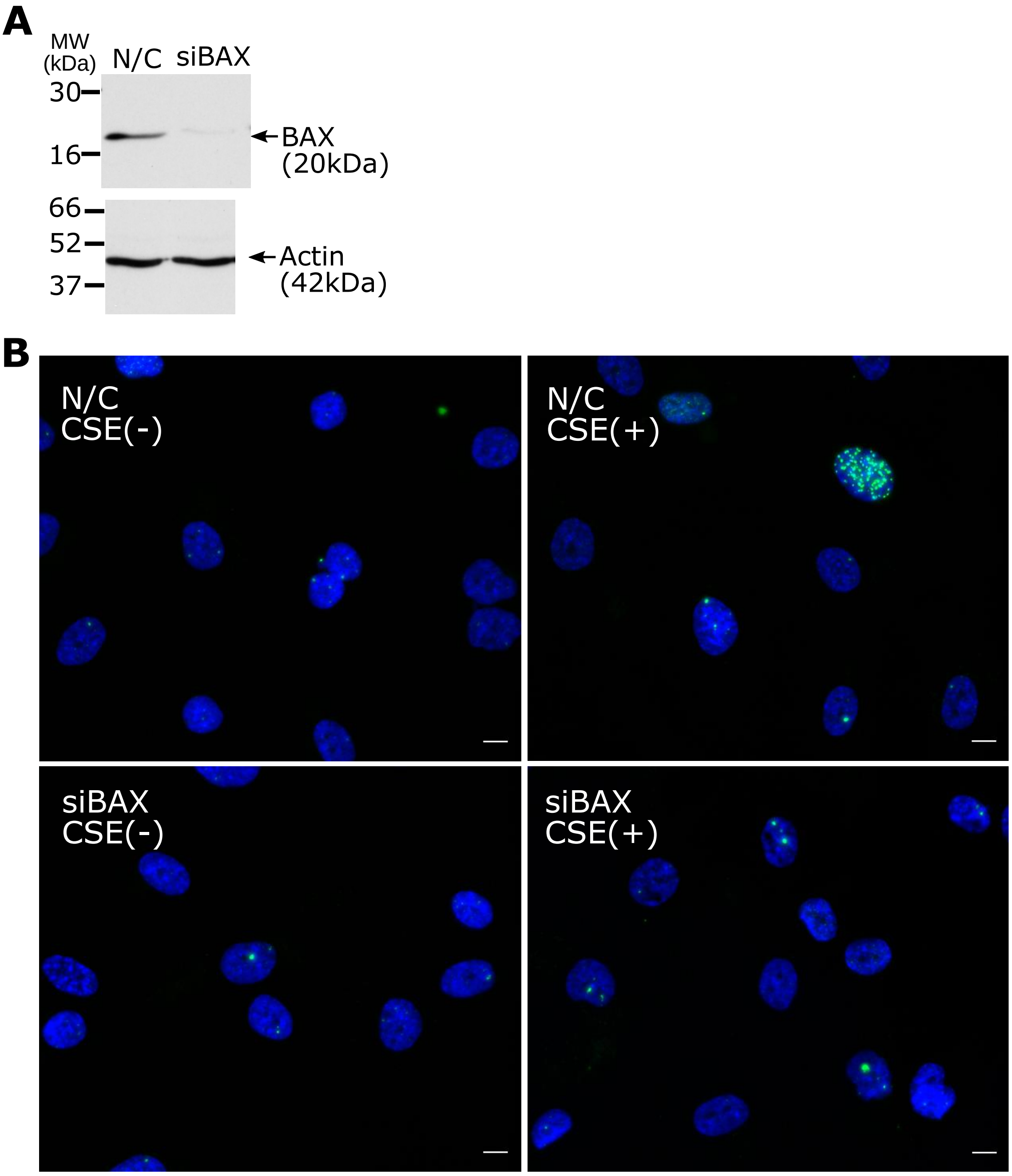

Supplementary Figure 3

(A) BAX expression after transfection with negative Control siRNA or BAX small interfering RNA (siRNA). Western blot analysis was performed with anti-BAX antibody. Arrow indicates BAX. (B) Low-power field images of the immunofluorescent staining of γH2AX in HUVECs transfected with siRNA against BAX (siBAX), or negative control siRNA (N/C) with or without CSE. Scale bar = 20 μm.

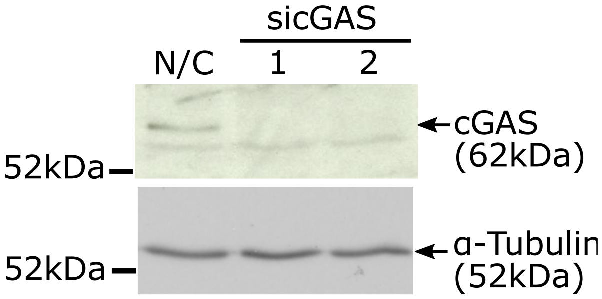

Supplementary Figure 4

cGAS expression after transfection with negative Control siRNA (N/C) or cGAS small interfering RNA (sicGAS). Western blot analysis was performed with anti-cGAS antibody. The arrow indicates the band corresponding to cGAS.

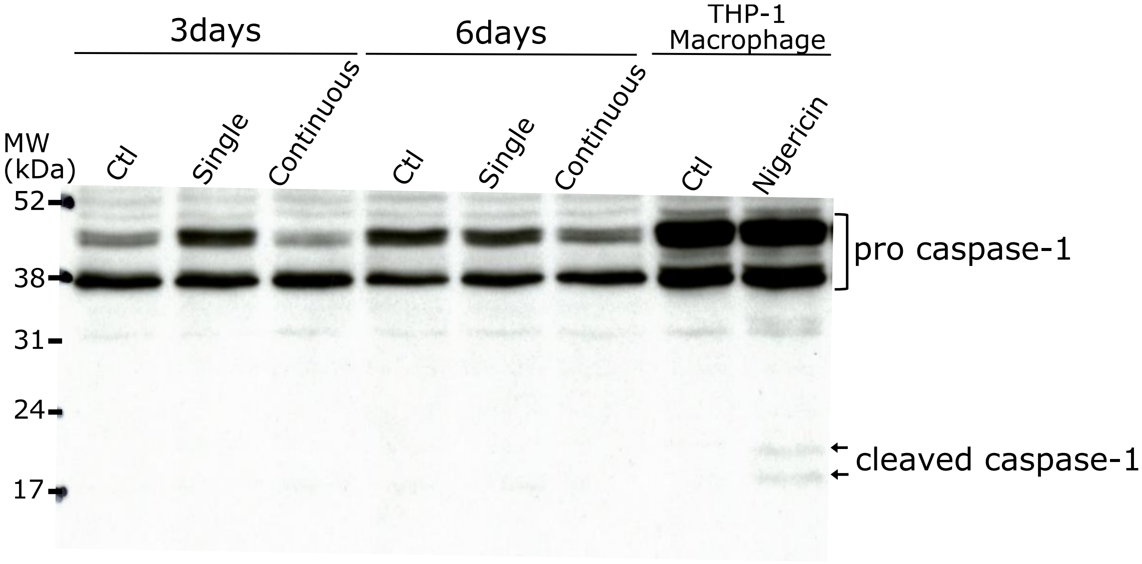

Supplementary Figure 5

Time course analysis of cytosolic caspase-1 levels by Western blotting. HUVECs were treated with CSE for 3 days or 6 days. As a positive control, THP-1 macrophages were treated with Nigericin.

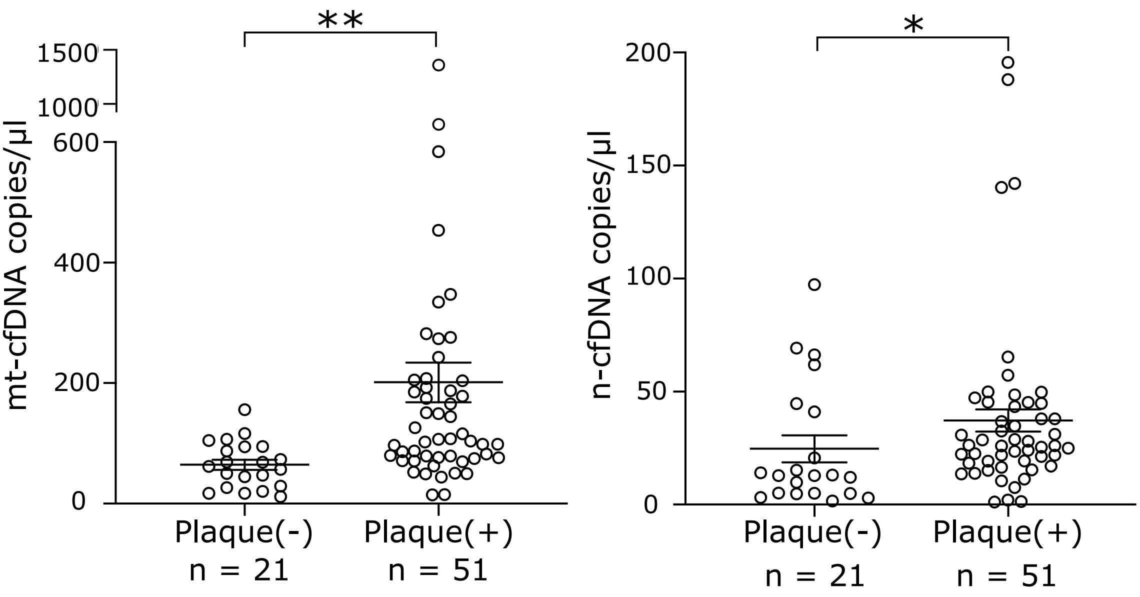

Supplementary Figure 6

The cfDNA copy number in normal subjects (Plaque [-]) and subjects with carotid plaques (Plaque [+]) in non-smokers. *P < 0.05, **P < 0.01 compared with Plaque (-).

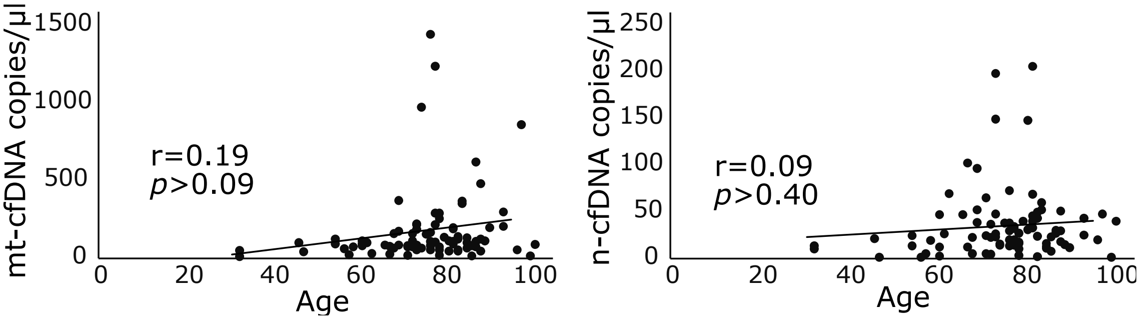

Supplementary Figure 7

Correlation of nuclear or mitochondrial cfDNA copy number with age. The correlation was analyzed using Pearson's correlation.

Supplementary Table 1. Primer Sequences

IL-6 forward primer (human)	AAGCCAGAGCTGTGCAGATGAGTA	qRT-PCR
IL-6 reverse primer (human)	TGTCCTGCAGCCACTGGTTC	qRT-PCR
IL-1a forward primer (human)	CTCAATTGTATGTGACTGCCCAAGA	qRT-PCR
IL-1a reverse primer (human)	TGGATGGGCAACTGATGTGAA	qRT-PCR
MCP-1 forward primer (human)	GCTCATAGCAGCCACCTTCATTC	qRT-PCR
MCP-1 reverse primer (human)	GGACACTTGCTGCTGGTGATTC	qRT-PCR
IFN-b forward primer (human)	AAACTCATGAGCAGTCTGCA	qRT-PCR
IFN-b reverse primer (human)	AGGAGATCTTCAGTTTCGGAGG	qRT-PCR
18s ribosomal RNA forward primer (human)	ACTCAACACGGGAAACCTCA	qRT-PCR
18s ribosomal RNA reverse primer (human)	AACCAGACAAATCGCTCCAC	qRT-PCR
HBB 5’	CAAACAGACACCATGGTGCACCTGACTCCTGAGGAGAAGTCTGCCGTTACTGCCCTGTGGGGCAAGGTG	PCR
HBB 3’	AACGTGGATGAAGTTGGTGGTGAGGCCCTGGGCAGGTTGGTATCAAGGTTACAAGACAGGTTT	PCR
NADH 15’	TCTTAACAACATACCCATGGCCAACCTCCTACTCCTCATTGTACCCATTCTAATCGCAATGGCATTCCT	PCR
NADH1 3’	AATGCTTACCGAACGAAAAATTCTAGGCTATATACAACTACGCAAAGGCCCCAACGTTGTA	PCR

IL-6 = Interleukin 6*, IL-1a* = Interleukin 1 a, *MCP-1* = monocyte chemoattractant molecule 1, *IFN-b* = Interferon-b, HBB = hemoglobin subunit beta, NADH1 = NADH dehydrogenase subunit 1

Supplementary Table 2. Sequences for small interfering RNA

Target gene	Target sequence (5'–3')
cyclic GMP-AMP synthase (human) (1)	GGAAGAAAUUAACGACAUUTT
cyclic GMP-AMP synthase (human) (2)	CCUUCUCUCACAUCGAAAATT
BAX (human)	GCGUCCACCAAGAAGCUGATT

Supplementary Table 3. Logistic regression analysis in non-smokers: associations between the presence of plaque and the log cfDNA and clinical profiles.

	Univariate			Multivariate		
	OR	95%CI	P value	OR	95% CI	P value
log mt-cfDNA	3.77	1.75-9.80	<0.0003	3.83	1.39-13.38	0.007
log n-cfDNA	1.71	1.05-2.91	0.03	1.07	0.57-2.03	0.84
Age	1.10	1.05-1.18	<0.0001	1.07	1.00-1.15	0.04
Male	2.88	0.96-9.95	0.06	1.32	0.31-5.87	0.71
BMI	1.00	0.91-1.11	0.96			
Hypertension	1.57	0.50-4.76	0.43			
Dyslipidemia	1.49	0.53-4.29	0.45			
Diabetes	1.04	0.37-2.95	0.94			
TG	1.01	0.99-1.02	0.26			
HDL-C	0.96	0.93-0.99	0.03	0.99	0.94-1.04	0.64
LDL-C	1.00	0.98-1.001	0.13			
HbA1c	0.96	0.67-1.01	0.13			
eGFR	0.96	0.93-0.99	0.02	0.98	0.93-1.02	0.32

Multivariate analysis was performed with the presence of plaque as binary dependent variables and with the log cfDNA, age, male, HDL-C, eGFR as covariates.
mt-cfDNA = mitochondrial cell-free DNA; n-cfDNA = nuclear cell-free DNA; BMI = body mass index; TG = Triglyceride; HDL-C = high-density lipoprotein cholesterol; LDL-C=low-density lipoprotein cholesterol; HbA1c = Hemoglobin A1c; eGFR = estimated glomerular filtration rate.

Supplementary Table 4. Baseline characteristics of study subjects divided into four groups

	Normal n=21	Mild n=28	Moderate n=17	Severe n=17
Age	64.5 (55.8-72.5)	70 (65.5-78)	78* (71-81)	78*† (75-84)
Male, n(%)	6 (28.6%)	15 (53.6%)	8 (47.1%)	12 (70.6%)
plaque thicknesses	0	2.5 (1.8-3.2)	6.9 (6-8.5)	13.1 (11.4-16.7)
HbA1c	5.9 (5.5-6.6)	6.2 (5.8-6.6)	6.3 (5.6-6.7)	6 (5.5-6.7)
TG	76 (61-103)	101 (67.8-125)	84 (74-126)	107 (90-124)
HDL-C	71 (63-77)	60 (53.8-64)	54 (49-66)	57 (45-66)
LDL-C	113 (95-142)	107 (85-122.3)	96 (81-105)	102 (92-138)
AST	21 (19-24)	20.5 (19-24.5)	22 (17-27)	25 (22-28)
ALT	16 (14-27)	19 (13.8-23)	15 (13-22)	22 (12-26)
eGFR	70.4 (62.2-73.5)	52.6 (45.4-64.6)	58.3 (50.8-70.6)	54.4 (44.7-69.5)
MBP	96 (81.6-102)	93 (86.7-98.8)	86.7 (83.7-94.7)	92.7 (84.3-99.7)
BMI	23 (20.8-26.6)	24.9 (22.7-28.4)	25.4 (24.3-27.1)	23.6 (22-26.2)
Hypertension, n(%)	13 (62%)	18 (64.3%)	16 (94.1%)	13 (76.5%)
Diabetes, n(%)	9 (42.9)	14 (50%)	10 (58.8)	9 (52.9%)
Dyslipidemia, n(%)	10 (47.6%)	14 (50%)	9 (52.9%)	11 (64.7%)
Atrial fibrillation , n(%)	0	3 (10.7%)	0	1 (5.8%)
Heart failure , n(%)	0	1 (3.6%)	0	0

Continuous data were expressed as median and interquartile range, and categorical data as number and ratio. Steel-Dwass test was performed for continuous data, and the Chi-square test was for categorical data. *p<0.05 vs Plaque(-), †p<0.05 vs Mild. HbA1c = Hemoglobin A1c; TG = Triglyceride; HDL-C = high-density lipoprotein cholesterol; LDL-C=low-density lipoprotein cholesterol; AST = aspartate transaminase; ALT = alanine aminotransferase; eGFR = estimated glomerular filtration rate; MBP = mean blood pressure; BMI = body mass index.
